# Altered Th17/Treg balance and therapeutic targeting of RORγ in primary focal hyperhidrosis

**DOI:** 10.3389/fimmu.2025.1656632

**Published:** 2025-10-17

**Authors:** Min Lin, Jianbo Lin, Quan Du, Yuanrong Tu, Jianfeng Chen

**Affiliations:** ^1^ Department of Thoracic Surgery, The First Affiliated Hospital of Fujian Medical University, Fuzhou, Fujian, China; ^2^ Department of Thoracic Surgery, National Regional Medical Center, Binhai Campus of the First Affiliated Hospital, Fujian Medical University, Fuzhou, Fujian, China

**Keywords:** immune imbalance, primary focal hyperhidrosis (PFH), Th17 cell, Treg cell, RORγ

## Abstract

**Background:**

Primary focal hyperhidrosis (PFH) significantly impacts patients’ physical and mental health, yet its underlying mechanisms remain unclear.

**Methods:**

This study involved 80 healthy controls and 60 patients each with primary palmar (PPH), craniofacial (PCH), or axillary hyperhidrosis (PAH). Peripheral blood mononuclear cells (PBMCs) were analyzed via flow cytometry to assess Th17 and Treg cell populations. Cytokine levels were measured in patient serum using ELISA, while sweat gland tissue from PAH patients underwent gene expression analysis. A pilocarpine-induced mouse model of hyperhidrosis was used to test SR2211, a RORγ inverse agonist.

**Results:**

PFH patients exhibited a disrupted Th17/Treg balance, with increased Th17 and decreased Treg cells across all subtypes compared to controls. Elevated IL-17 and IL-6 and reduced IL-10 and TGF-β1 levels were observed in PFH serum. Sweat glands showed increased *RORγt* and decreased *FOXP3* expression. In mice, SR2211 treatment reduced sweat secretion, secretory granules, and serum acetylcholine. It also lowered Th17 infiltration, serum IL-17/IL-6, and IL-17A expression in sweat glands.

**Discussion:**

PFH is associated with a Th17/Treg immune imbalance. SR2211 alleviated hyperhidrosis and Th17-related inflammation in mice, highlighting the potential of targeting the RORγ–Th17 axis as a therapeutic strategy for PFH.

## Introduction

Primary focal hyperhidrosis (PFH) is a medical condition characterized by excessive and unpredictable sweating, specifically targeting localized areas of the body, such as the palms, soles of the feet, underarms, or face (1–3). Typically manifesting during adolescence or early adulthood, primary focal hyperhidrosis can significantly impact an individual’s daily life, social interactions, and emotional well-being (4, 5). While the exact cause of primary focal hyperhidrosis is not fully understood, it is believed to have a genetic component, as it often runs in families (6). Stress and anxiety can exacerbate the symptoms, making the condition more pronounced in high-pressure situations (7, 8). Managing primary focal hyperhidrosis involves a range of treatment options, tailored to individual’s needs and severity of symptoms (9, 10).

Non-surgical treatments for PFH encompass various options, including topical antiperspirants, oral anticholinergics, botulinum toxin injections, and microwave pyrolysis (11). While these treatments yield favorable short-term results, they are prone to relapse, and some may entail side effects such as muscle weakness and localized pain (12). Among the treatment methods available, thoracic sympathectomy stands as the most effective approach for treating primary palmar hyperhidrosis (PPH), extending its efficacy to hyperhidrosis affecting other regions (12, 13). Nevertheless, the procedure’s high incidence of postoperative compensatory hyperhidrosis significantly impacts patients’ quality of life, and in severe cases, may even trigger mental health issues (14).

The finely regulated neuro-endocrine-immune network enables the immune system to perceive stimuli that the nervous system cannot directly sense and modulate nervous system functions (15). Sympathetic nerves, which are widely distributed throughout the body, including lymphoid organs, form synaptic connections with ganglia along the sympathetic chain or lateral processes, with autonomic nerve fibers found in the vasculature and parenchyma of these organs, containing numerous T cells (16). Curiously, despite the extensive research on primary focal hyperhidrosis, no reports have yet analyzed the specific changes in the T cell immune system in patients with this condition (17). Further investigation in this area may shed light on additional aspects of PFH pathology and potential treatment avenues (18). Therefore, the purpose of this work is to study the balance changes of Th17/Treg T cells in the peripheral blood of PFH patients, so as to provide more physiological basis for the treatment of PFH.

## Methods

### Participants

The present study included 80 healthy controls and 60 cases each of primary palmar hyperhidrosis (PPH), primary craniomofacial hyperhidrosis (PCH), and primary axillary hyperhidrosis (PAH). All participants were of Han ethnicity and were recruited from the First Affiliated Hospital of Fujian Medical University, with age and gender matched between the two groups. Both groups also met the same inclusion and exclusion criteria, as outlined below:

Inclusion criteria: Participants aged 18 years or older with informed consent.

Exclusion criteria: Individuals with significant damage to vital organs such as heart, liver, or kidneys; those with infectious diseases or autoimmune conditions; individuals on long-term steroid or other immunosuppressive medication; and patients with tumors were excluded from the study.

The study was approved by the Ethics Committee of First Affiliated Hospital of Fujian Medical University ([2021]466), and written informed consent was derived from the participants.

### Isolation of peripheral blood mononuclear cells

All participants’ fasting blood was collected for the isolation of PBMCs using aseptic techniques. Firstly, 1 ml of Ficoll was added to a 15 ml centrifuge tube, followed by the addition of 1 ml of freshly collected anticoagulated peripheral blood mixed with an equal amount of PBS. Carefully added to the top of the Ficoll layer, the liquid interface was maintained as clear as possible. The mixture was centrifuged at 400×g for 15 minutes at room temperature. After centrifugation, four layers were formed from top to bottom: the plasma layer, mononuclear cell layer, transparent separation fluid layer, and blood cell layer. The second cloudy layer containing the target cells (mononuclear cells) was carefully aspirated and transferred to a centrifuge tube containing PBS. The cells were thoroughly mixed and centrifuged at 2000 rpm for 10 minutes. After two additional washes with PBS, PBMCs were obtained. The PBMCs were suspended in RPMI1640 culture medium, and the cell concentration was adjusted to 1-2×10^6^/ml. For Treg labeling, 500 μl of PBMCs was taken, and for Th17 cell labeling, 600 μl was placed in a 24-well cell culture plate. Stimulation-blocking agents, PMA, and BFA, were added, and the cells were cultured at 37°C, 5% CO_2_ in a cell culture incubator for 5 hours.

### The proportion of Th17 cells

Cells were resuspended, and 100 μl of the cell suspension was aliquoted into separate 1.5 ml EP tubes, including a blank tube, a compensation tube, and a sample tube. Surface antibodies CD3-FITC (85-11-0037-42, eBioscience) and CD8a-APC (85-17-0086-42, eBioscience) (5 μl each) were added to each tube. Incubation was performed at room temperature in the dark for 30 minutes, followed by centrifugation at 400×g for 5 minutes. The supernatant was discarded, and cells were resuspended. Fixative permeabilization reagent A (100 μl) was added and incubated at room temperature for 15 minutes. Flow cytometry buffer was added, mixed, and centrifuged at 400×g for 5 minutes. The supernatant was discarded, and cells were resuspended. Fixative permeabilization reagent B and IL-17-PE antibody (85-12-7179-42, eBioscience) (100 μl each) were added and incubated at 4°C in the dark for 30 minutes. Flow cytometry buffer was added, mixed, and centrifuged at 400×g for 5 minutes. The supernatant was discarded, and 4% paraformaldehyde (100 μl) was added. Incubation was performed at 4°C in the dark for 15 minutes. After washing, cells were resuspended in 300 μl of PBS and stored at 4°C in the dark for subsequent flow cytometric analysis. The gating strategy used for Th17 cells has been provided in **Supplementary Figure S1**.

### The proportion of Treg cells

Cells were resuspended, and 100 μl of the cell suspension was placed in each of the tubes (blank, compensation, and sample). Surface antibodies CD4-FITC (85-11-0047-42, eBioscience) and CD25-PE (85-12-0259-42, eBioscience) (5 μl each) were added to each tube and incubated for 25 minutes at room temperature in the dark. After centrifugation, flow cytometry buffer was added, and the supernatant was discarded. Fixed and permeabilization reagent D (1 ml) was added and incubated in the dark at room temperature for 40 minutes. Diluted washing buffer E (2 ml) was added after centrifugation, and cells were resuspended after discarding the supernatant.

Intracellular antibody Foxp3-APC (85-17-4776-42, eBioscience) (5 μl) was added and incubated at room temperature in the dark for 40 minutes. After centrifugation, cells were washed with washing buffer E (2 ml), and the supernatant was discarded. 4% paraformaldehyde (100 μl) was added and incubated at 4°C in the dark for 15 minutes. Following two washes, cells were resuspended in 300 μl of PBS and stored at 4°C in the dark for subsequent flow cytometric analysis. The gating strategy was similar to that used for Th17 cells. Similarly, PBMCs were gated based on FSC/SSC, and doublets were excluded. T lymphocytes were identified by gating CD4+ cells, followed by gating Treg cells based on Foxp3 and CD25 expression. The gating strategy for Treg cells followed the same principles as Th17 cells (**Supplementary Figure S1**).

### Collection of sweat gland tissue

At our hospital, sweat gland tissue from patients with PFH was collected through a single-port axillary surgery. A 2 mm wide and 5 mm long full-thickness skin incision was made, and the sweat gland tissue was extracted. Surrounding tissues of a similar volume were collected as the control group. All cases included in the study had no history of palmar hyperhidrosis, axillary hyperhidrosis, or a family history of such conditions. Additionally, there were no reported histories of bromhidrosis (foul-smelling sweat) in any of the patients. The experimental protocol was carried out with the patients’ written consent.

### RT-PCR assay

The RT-PCR assay was performed to analyze the gene expression levels of FOXP3, RORγt, and β-actin in the samples. Total RNA was extracted from the desired samples, and cDNA was synthesized using a reverse transcription kit. Specific forward and reverse primers for each target gene (FOXP3, RORγt, and β-actin) were used. Additionally, the Primer-BLAST tool available on the NCBI website was used to evaluate the specificity of the primers. PCR reactions were set up with the cDNA templates and the respective primer pairs, followed by thermal cycling conditions for amplification. The PCR products were visualized through agarose gel electrophoresis, and the bands were analyzed to quantify gene expression levels relative to the reference gene β-actin. The primers were as below:

FOXP3: F: TTTCTGTCAGTCCACTTCACCA

R: CCAGCAGGTCTGAGGCTTTG;

RORγt: F: GTGGGGACAAGTCGTCTGG

R: AGTGCTGGCATCGGTTTCG;

β-actin: F: TGGCACCCAGCACAATGAA

R: CTAAGTCATAGTCCGCCTAGAAGCA

### Enzyme-linked immunosorbent assay

The concentrations of IL-17 (EH3267, sensitivity: 18.75 pg/ml, CV (coefficient of variation) of inter-assay: 4.94%, CV of intra-assay: 5.27%), IL-10 (EH0173, sensitivity: 4.688 pg/ml, CV of inter-assay: 5.05%, CV of intra-assay: 5.19%), IL-6 (EH0201, sensitivity: 2.813 pg/ml, CV of inter-assay: 4.66%, CV of intra-assay: 4.87%) and TGF-β1 (EH0287, sensitivity: 18.75 pg/ml, CV of inter-assay: 5.53%, CV of intra-assay: 5.92%) in the serum of patients were detected by ELISA method (Fine Test, Wuhan, China). Representative standard curves used to calculate cytokine concentrations are provided in **Supplementary Figure S2**.

### Western blotting assay

Protein expression of RORγt, FOXP3, IL-17A, and GAPDH in sweat gland tissues was analyzed by Western blotting. Briefly, tissues were lysed in RIPA buffer supplemented with protease inhibitors, and protein concentration was determined by BCA assay. Equal amounts of protein (30 μg) were separated by SDS-PAGE and transferred to PVDF membranes. Membranes were blocked with 5% non-fat milk and incubated overnight at 4°C with the following primary antibodies: RORγt (ab113434, Abcam), FOXP3 (ab215206, Abcam), IL-17A (ab79056, Abcam), and GAPDH (ab8245, Abcam). After washing, membranes were incubated with HRP-conjugated secondary antibodies for 1 hour at room temperature. Protein bands were visualized using enhanced chemiluminescence (ECL) reagents and quantified with ImageJ software.

### Animal model and pharmacological intervention

SR2211, a potent and selective synthetic modulator of RORγ that functions as an inverse agonist, was used for *in vivo* intervention (19). According to the manufacturer’s data, SR2211 functions as an inverse agonist of RORγ (Ki = 105 nM, IC50 ~320 nM) with minimal off-target activity (>10 μM against RORα, RORβ, TRK, and BRAF). SR2211 was purchased from MedChemExpress (MCE, Cat# HY-16998).

Animal studies were approved by the First Affiliated Hospital of Fujian Medical University. Hyperhidrosis was induced by intraperitoneal injection of pilocarpine hydrochloride at a dose of 5 mg/kg body weight. Five minutes after pilocarpine administration, the appearance of black sweat dots on the plantar surface was photographed to quantify sweat secretion. For intervention, SR2211 was administered intraperitoneally at 20 mg/kg once daily for seven consecutive days prior to hyperhidrosis induction. The dosage and route of administration were based on previously published methods (20). Two hours post-induction, mice were euthanized, and blood and sweat gland tissues were collected. Serum acetylcholine (Ach) levels were measured using a commercially available ELISA kit (Acetylcholine ELISA Kit, OKEH02568; Aviva Systems Biology, San Diego, CA), according to the manufacturer’s instructions. Flow cytometry was performed on whole blood to assess the proportion of Th17 T cells. Serum IL-17 and IL-6 levels were analyzed by ELISA, and protein expression of IL-17A in sweat gland tissues was evaluated via Western blotting.

### Statistical analysis

The data obtained in the study were subjected to statistical analysis using SPSS 15.0 software. The sample size was calculated based on the following equation: N = φ^2^(ΣSi^2^/g)/[Σ( Xi - X)^2^/(g-1)], N represents the sample size of each group, g is the number of groups, X and Si are the mean and standard deviation of each group, respectively. Estimates of effect size and standard deviation were based on the existing literature and our previous experiences. To calculate the power of analysis we assumed α = 0.05 and β = 0.2. For continuous data, t-tests were performed for comparisons between two groups, while for categorical data, χ² tests were conducted. For multiple comparisons, Brown-Forsythe ANOVA test followed by Dunnett’s T3 multiple comparisons test was employed. During the analysis, if the P-value was less than 0.05 (p< 0.05), it was considered as indicating a significant difference.

## Results

### Demographic and clinical characteristics

A comprehensive cohort of 240 participants was enrolled in the current study, encompassing 80 individuals designated as healthy controls (HC) and 60 cases each of PPH, PCH, and PAH. Notably, no statistically significant disparities emerged in terms of age (p=0.297) and gender distribution (p=0.336) between the healthy control group and the PFH group (**Table 1**).

**Table 1:** Demographic and clinical characteristics of patients with primary focal hyperhidrosis (PFH) and healthy controls (HC) enrolled in the study.

Characteristics	Study group		p
	HC (n = 80)	PFH (n = 180)	
Age	28.85 ± 5.92	29.37 ± 7.46	0.297
Gender			
Male	46 (57.5%)	115 (63.9%)	0.336
Female	34 (42.5%)	65 (36.1%)

Values were expressed as n (percentage, %) or mean ± SD. p values were derived from Mann-Whitney test. Fisher’s exact test was used for assessing distribution of observations.

### PFH patients exhibit an altered Th17/Treg balance in peripheral blood

**Figure 1:**
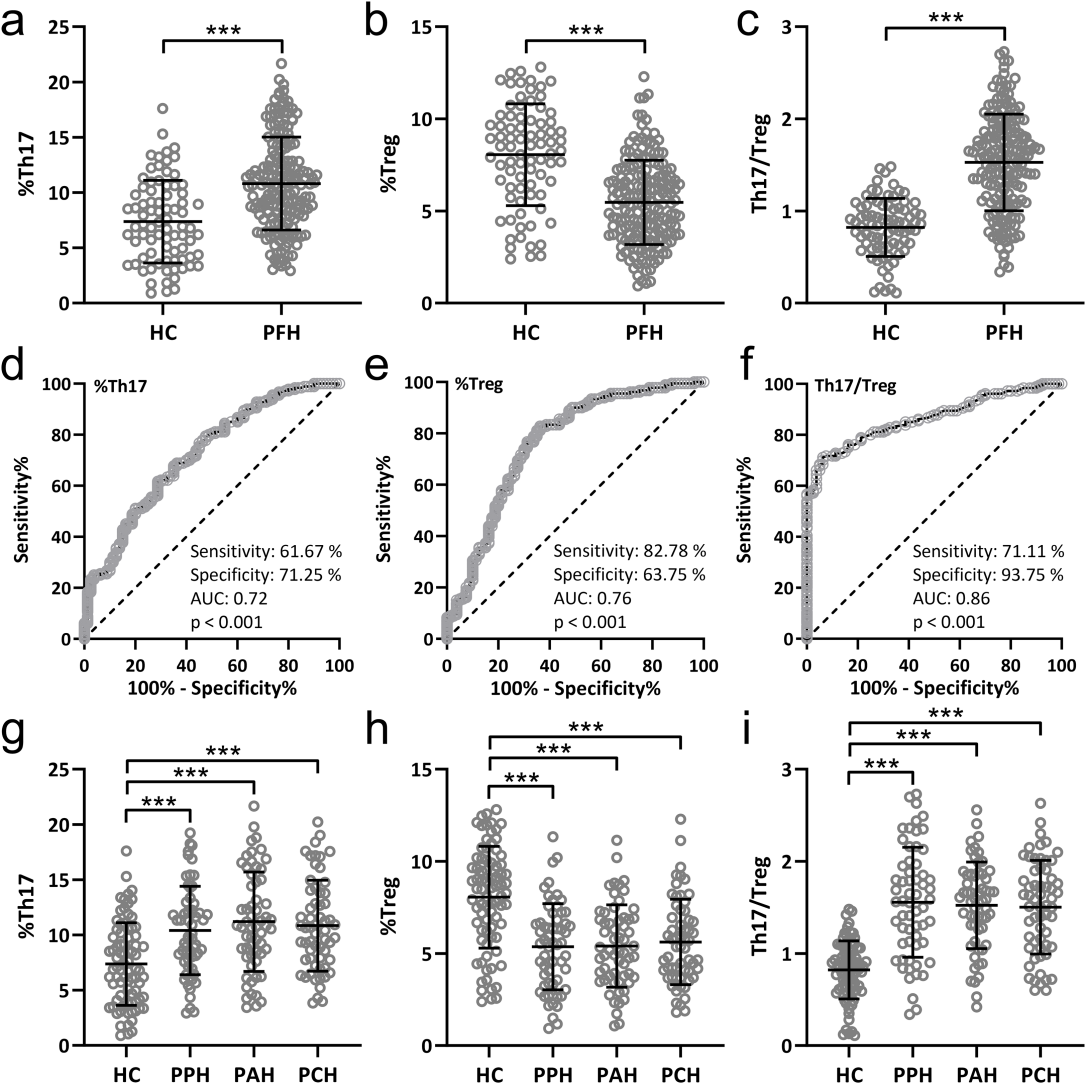
Comparisons of Th17 **(A)**, Treg **(B)** lymphocyte subpopulations and Th17/Treg ratio **(C)** in peripheral blood from patients with primary focal hyperhidrosis (PFH, n = 180) and healthy controls (HC, n = 80). Data were shown as mean ± SD. ***p < 0.001. Unpaired t test with Welch’s correction. ROC analysis of Th17 **(D)**, Treg **(E)** lymphocyte subpopulations and Th17/Treg ratio **(F)** in peripheral blood to identify primary focal hyperhidrosis patients from healthy controls. Comparisons of Th17 **(G)**, Treg **(H)** lymphocyte subpopulations and Th17/Treg ratio **(I)** in peripheral blood from healthy controls (n = 80), primary palmar hyperhidrosis patients (n = 60), primary axillary hyperhidrosis patients (n = 60) and primary cranimofacial hyperhidrosis patients (n = 60). The data were presented with mean ± SD. ***p < 0.001 from Brown-Forsythe ANOVA test followed by Dunnett’s T3 multiple comparisons test.

The equilibrium of the Th17/Treg cell ratio within the peripheral blood of individuals afflicted with PFH was meticulously explored and juxtaposed against that of 80 healthy controls, alongside a pool of 180 PFH patients. The findings underscored that PFH patients manifested an elevated proportion of Th17 cells (**Figure 1A**), accompanied by a decreased proportion of Treg cells (**Figure 1B**) within the peripheral blood milieu. This dysregulation ultimately culminated in a substantial augmentation of the Th17/Treg ratio (**Figure 1C**). Furthermore, receiver operating characteristic (ROC) analysis unmasked the diagnostic potential of Th17 and Treg lymphocyte subpopulations, as well as the Th17/Treg ratio within the peripheral blood, in the context of PFH. Th17 exhibited a sensitivity of 61.67%, a specificity of 71.25%, an area under the curve (AUC) of 0.72, and p-value < 0.001 (**Figure 1D**). Treg demonstrated a sensitivity of 82.78%, a specificity of 64.75%, an AUC of 0.76, and p-value < 0.001 (**Figure 1E**). Most notably, the Th17/Treg ratio emerged as particularly indicative, with a sensitivity of 71.11%, a specificity of 93.75%, an AUC of 0.86, and p-value < 0.001 (**Figure 1F**). This rigorous evaluation accentuates the diagnostic utility of Th17, Treg lymphocyte subpopulations, and the Th17/Treg ratio within peripheral blood as discerning markers for PFH.

### Th17/Treg imbalance is consistent across PFH clinical subtypes

The comparisons of Th17 (**Figure 1G**), Treg (**Figure 1H**) lymphocyte subpopulations, and Th17/Treg ratio (**Figure 1I**) in peripheral blood among 80 healthy controls, 60 patients with PPH, 60 patients with PCH, and 60 patients with PAH. The results suggest that there were no significant differences in Th17/Treg cell ratio among different subtypes of PFH patients. However, all PFH subtypes showed significant differences compared to the healthy controls.

### PFH patients display dysregulated cytokine profiles

**Figure 2:**
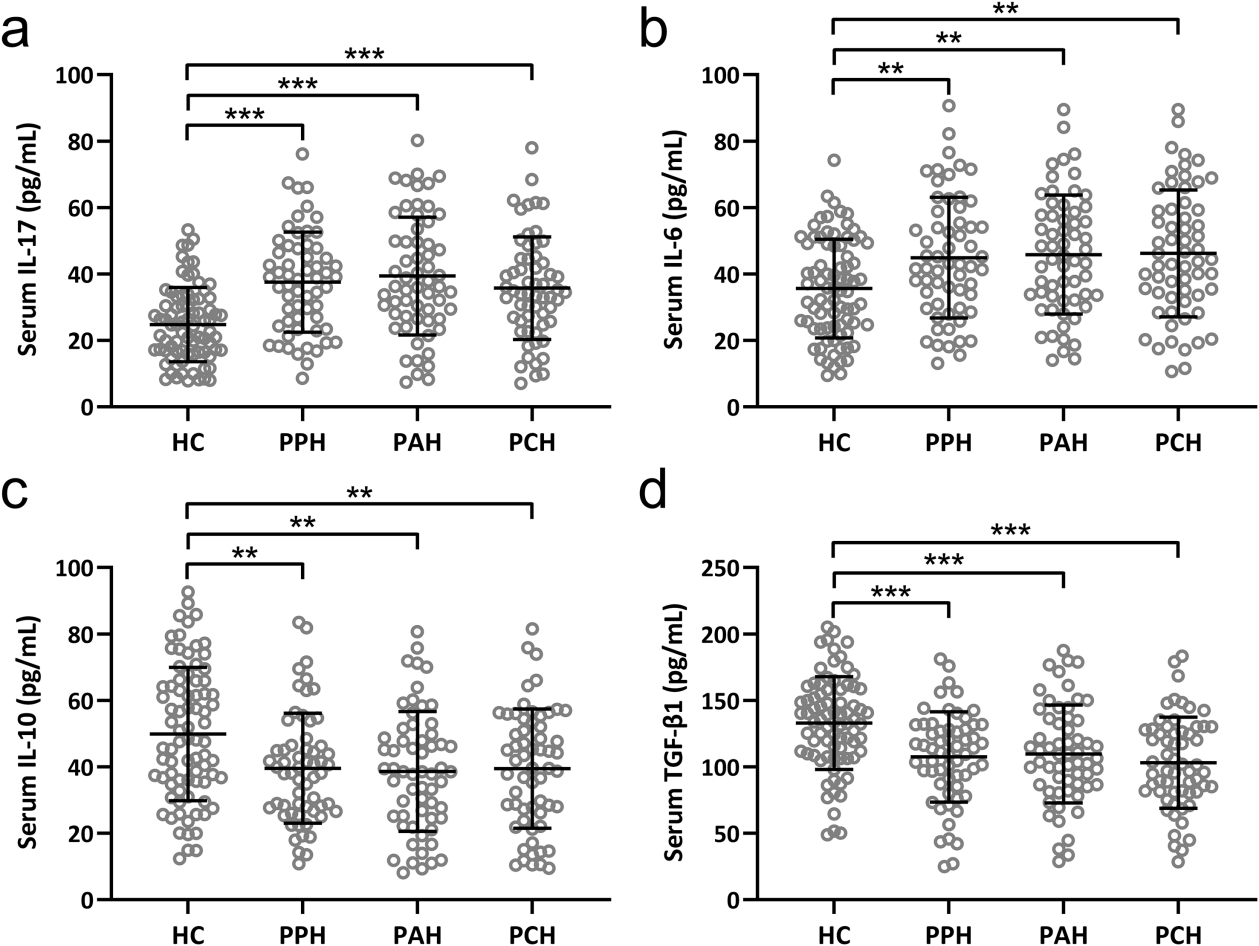
Comparisons of serum IL-17 **(A)**, IL-6 **(B)**, IL-10 **(C)** and TGF-β1 **(D)** among healthy controls (n = 80), primary palmar hyperhidrosis patients (n = 60), primary axillary hyperhidrosis patients (n = 60) and primary cranimofacial hyperhidrosis patients (n = 60). The data were presented with mean ± SD. **p < 0.01, ***p < 0.001 from Brown-Forsythe ANOVA test followed by Dunnett’s T3 multiple comparisons test.

We carried out the concentration detection of the relevant cytokines of two kinds of cells. Notably, IL-17 and IL-6, recognized as Th17-associated cytokines (21), along with IL-10 and TGF-β1, known to be Treg-associated cytokines (22), underwent careful scrutiny. The discerned serum levels of IL-17 and IL-6 were notably and significantly escalated across all three PFH subtypes in comparison to the healthy control cohort (**Figures 2A, B**). Conversely, a strikingly converse pattern emerged for IL-10 and TGF-β1, with both these cytokines registering marked reductions in the serum of patients encompassing all three PFH subtypes as opposed to healthy controls (**Figures 2C, D**). These findings mirror previous outcomes, further underscoring the prevailing Th17/Treg imbalance within PFH patients.

### RORγt is upregulated and FOXP3 is downregulated in sweat gland tissues of PFH patients

**Figure 3:**
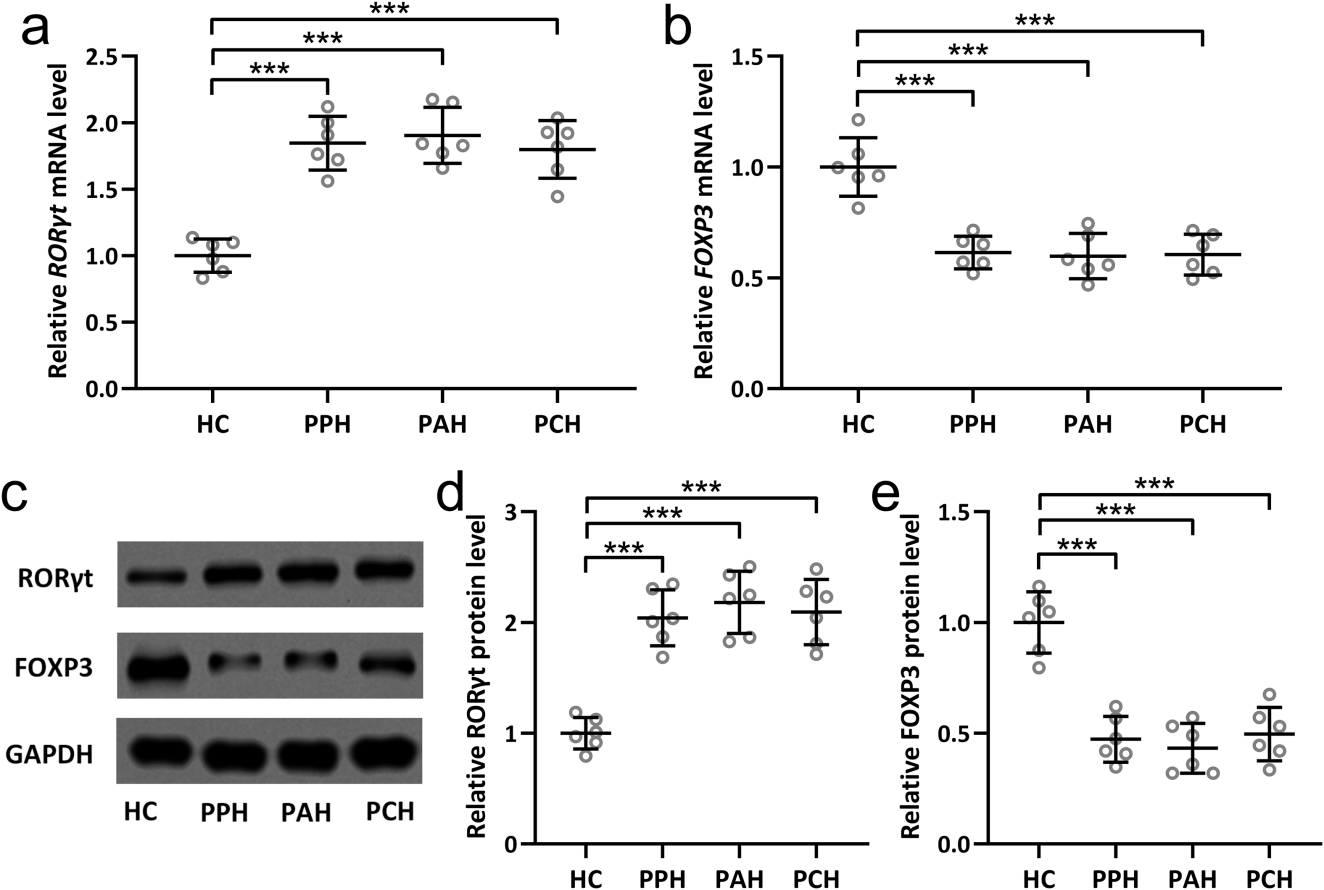
Expression changes of RORγt and FOXP3 in sweat gland tissues of primary focal hyperhidrosis patients. The mRNA levels of RORγt **(A)** and FOXP3 **(B)** in sweat gland tissues from healthy controls, primary palmar hyperhidrosis patients, primary axillary hyperhidrosis patients and primary cranimofacial hyperhidrosis patients were measured by qRT-PCR. n = 6 for each group. The protein levels of RORγt and FOXP3 in sweat gland tissues homogenate from healthy controls, primary palmar hyperhidrosis patients, primary axillary hyperhidrosis patients and primary cranimofacial hyperhidrosis patients were measured by Western blotting **(C)**. β-actin was used as a loading control and the expressions were normalized to HC **(D, E)**. n = 6 for each group. The data were presented with mean ± SD. ***p < 0.001 from Brown-Forsythe ANOVA test followed by Dunnett’s T3 multiple comparisons test.

Th17 and Treg cells are characterized by the selective expression of distinct transcriptional factors. RORγt is characteristic for Th17 cells (23), whereas FOXP3 is characteristic for Treg cells (24). The current investigation ventured into scrutinizing the expression variations of RORγt and FOXP3 within sweat gland tissues extracted from PFH patients. The results unveiled substantial elevation at both the mRNA and protein levels of RORγt (**Figures 3A, C, D**) across all three PFH subtypes in comparison to the healthy control group. Conversely, for FOXP3, both mRNA and protein levels exhibited noteworthy diminutions within sweat gland tissues across all three PFH subtypes relative to healthy controls (**Figures 3B, C, E**). Collectively, these findings reinforce the assertion that PFH is intrinsically associated with a discernible Th17/Treg immune imbalance. This comprehensive evaluation contributes crucial insights into the intricate immunological underpinnings of PFH, underscoring the pivotal role played by Th17 and Treg cells alongside their associated cytokine profiles in shaping the pathophysiological landscape of this condition.

### SR2211 reduces sweat hypersecretion in a mouse model of hyperhidrosis

**Figure 4:**
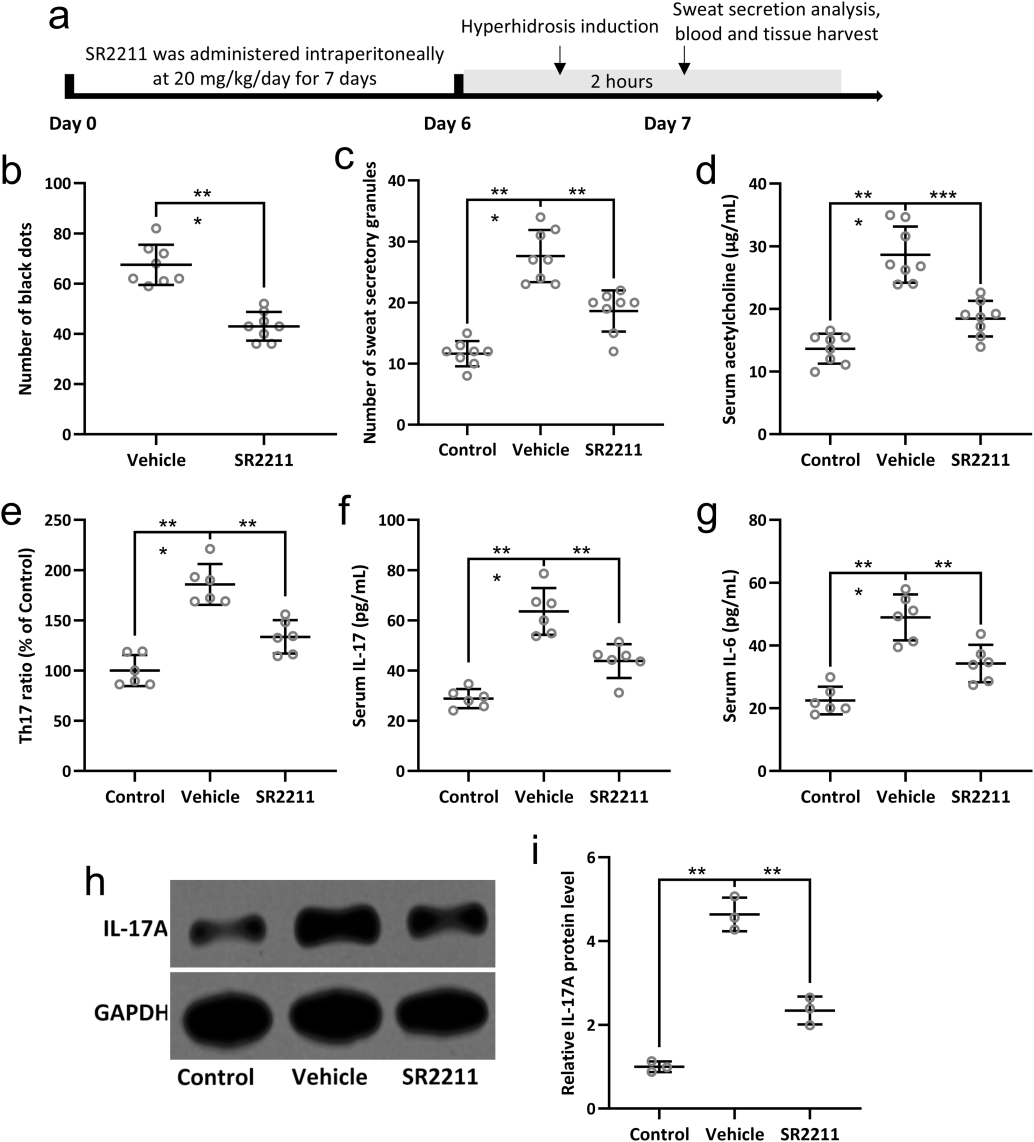
SR2211, the inverse agonist of RORγ, attenuated the sweat secretion and Th17 lymphocyte related inflammatory response in hyperhidrosis mice. Mice were administered with vehicle or SR2211 for one week before the induction of hyperhidrosis, as shown in **(A)**. The number of black dots were calculated **(B)**. The number of sweat secretory granules were counted **(C)** and the concentration of acetylcholine in serum was detected by ELISA **(D)**. Th17 lymphocyte subpopulations in peripheral blood of the mice were compared **(E)**. The serum levels of IL-17 **(F)** and IL-6 **(G)** were measured by ELISA. Western blotting was used to detect the protein expression of IL-17A in the sweat gland of hyperhidrosis mice **(H)**. GAPDH was used as a loading control and the expressions were normalized to control **(I)**. The data were presented with mean ± SD. n = 8 for each group. **p < 0.01, ***p < 0.001 from Brown-Forsythe ANOVA test followed by Dunnett’s T3 multiple comparisons test.

To evaluate the therapeutic effect of SR2211, an inverse agonist of RORγ (25), on sweat secretion in a mouse model of hyperhidrosis, mice were intraperitoneally administered with SR2211 (20 mg/kg) or vehicle daily for one week prior to pilocarpine-induced hyperhidrosis (**Figure 4A**). Pilocarpine hydrochloride (5 mg/kg) was injected intraperitoneally to induce sweat secretion, and the sweat output was quantified by counting black dots formed on the paw surface (26). As shown in **Figure 4B**, the number of black dots was significantly increased in hyperhidrosis model mice compared to the vehicle-treated control group, confirming successful model establishment. Notably, pretreatment with SR2211 markedly reduced the number of black dots (p < 0.001), indicating that SR2211 effectively attenuated excessive sweat secretion.

Further histological analysis revealed that the number of sweat secretory granules in the sweat glands was significantly decreased following SR2211 treatment compared with hyperhidrosis model mice (**Figure 4C**, p < 0.01), supporting the inhibitory effect of SR2211 on sweat gland activity. Additionally, ELISA of serum acetylcholine concentrations demonstrated a significant reduction in acetylcholine levels in the SR2211-treated group compared with the untreated hyperhidrosis group (**Figure 4D**, p < 0.01), suggesting that SR2211 may alleviate hyperhidrosis by modulating cholinergic signaling. Collectively, these results demonstrate that SR2211 ameliorates hyperhidrosis-like symptoms in mice, as evidenced by decreased sweat secretion, reduced secretory granules, and lower serum acetylcholine levels.

### SR2211 attenuates Th17-associated inflammation in hyperhidrosis mice

To investigate the immunomodulatory effects of SR2211 on Th17-related inflammation in hyperhidrosis, we analyzed peripheral blood lymphocyte subpopulations and pro-inflammatory cytokines. Flow cytometry revealed a significant increase in Th17 lymphocyte proportions in hyperhidrosis model mice compared with vehicle-treated controls. Importantly, SR2211 treatment markedly reduced the proportion of circulating Th17 cells (**Figure 4E**, p < 0.01), indicating a suppressive effect on Th17 cell differentiation or expansion. Consistent with these findings, ELISA analysis showed elevated serum levels of IL-17 and IL-6 in hyperhidrosis mice, both of which were significantly reduced following SR2211 administration (**Figures 4F, G**, p < 0.01 and p < 0.001, respectively). These results suggest that SR2211 dampens systemic Th17-driven inflammatory responses.

To further validate the local inflammatory status within sweat glands, IL-17A protein expression was examined by Western blotting. IL-17A expression in sweat gland tissues was upregulated in hyperhidrosis mice but significantly decreased after SR2211 treatment (**Figures 4H, I**, p < 0.01), while GAPDH served as a loading control. Collectively, these data demonstrate that SR2211 effectively suppresses Th17 cell-associated inflammatory responses both systemically and locally in hyperhidrosis mice, aligning with clinical observations of Th17 involvement in hyperhidrosis pathophysiology.

## Discussion

The available therapies can be categorized into non-surgical and surgical options. Non-surgical treatments for PFH encompass a variety of approaches. Topical antiperspirants containing aluminum chloride are commonly used and are effective for mild cases of hyperhidrosis (27). They work by blocking sweat ducts and reducing sweat production. Oral anticholinergic medications are another option that can reduce sweating by targeting the sweat glands’ nerve signals (28). However, they may have side effects like dry mouth, constipation, and blurred vision. The most effective surgical approach is thoracic sympathectomy (27). Thoracic sympathectomy is currently the most effective treatment for PPH This procedure involves cutting or clamping the sympathetic nerves responsible for triggering excessive sweating (29). It is highly effective for treating primary palmar hyperhidrosis and can also be used for other localized areas. However, one potential side effect is compensatory hyperhidrosis, where excessive sweating occurs in other parts of the body after the surgery (29). Therefore, the discovery of the pathogenesis of PFH and the exploration of related treatment methods are urgently needed.

In this study, we investigated the Th17/Treg cell balance in peripheral blood from patients with PFH. The results revealed a significant alteration in Th17/Treg cell ratio in PFH patients compared to healthy controls, indicating an immune imbalance in the disease. Th17 cells, which are associated with inflammatory responses, were found to be increased, while Treg cells, which play a role in immune regulation and tolerance, were decreased in PFH patients. From our perspective, this Th17/Treg imbalance is likely not a simple cause or consequence, but rather part of a bidirectional feedback loop: enhanced Th17 responses and impaired Treg activity may drive local inflammation and sweat gland dysfunction, while pathological changes in sweat glands may in turn exacerbate the imbalance. These findings suggest a potential dysregulation of the immune system in PFH, which may contribute to the pathogenesis of the condition. We believe that the novelty of the study lies in its investigation of the Th17/Treg balance in patients with PFH, a medical condition characterized by excessive sweating in localized areas of the body. Our study explores the immune dysregulation in PFH patients and its potential role in the pathology of the disease.

Our study also compared the Th17/Treg cell ratio among different subtypes of PFH patients, including PPH, PCH, and PAH. Interestingly, no significant differences in Th17/Treg cell ratio were observed among these subtypes, indicating a similar immune imbalance across different localized areas of hyperhidrosis. However, all PFH subtypes showed significant differences compared to healthy controls, highlighting the potential role of Th17/Treg imbalance as a common feature in PFH pathophysiology. Furthermore, we investigated the cytokine profile in PFH patients, specifically focusing on Th17-associated cytokines (IL-17 and IL-6) and Treg-associated cytokines (IL-10 and TGF-β1). Consistent with the changes observed in Th17/Treg cell ratio, PFH patients exhibited elevated levels of IL-17 and IL-6 and decreased levels of IL-10 and TGF-β1 in the serum. Although additional cytokines such as IL-21 and IL-22 could provide a broader picture of Th17 activity, we prioritized core cytokines to maintain a focused design. The lack of IL-21/IL-22 data is a limitation, which we now explicitly acknowledge. These findings support the notion of an immune dysregulation involving Th17 and Treg cells in PFH.

To gain further insights into the molecular mechanisms underlying the Th17/Treg imbalance in PFH, we examined the expression of key transcription factors (RORγt for Th17 and FOXP3 for Treg) in sweat gland tissues from PFH patients. Our results demonstrated a significant upregulation of RORγt and downregulation of FOXP3 at both mRNA and protein levels in all three PFH subtypes. These findings provide additional evidence for the involvement of Th17 and Treg cells in the pathogenesis of PFH. Moreover, our parallel findings between peripheral blood (cell ratios, cytokines) and sweat gland tissues (transcription factors, protein expression) suggest that systemic immune alterations align, at least in part, with the local tissue microenvironment. However, peripheral blood cannot fully capture the complexity of local immune regulation, which remains a limitation. The altered Th17/Treg balance in PFH patients may have important clinical implications. Th17 cells are known to promote inflammation, whereas Treg cells play a crucial role in suppressing excessive immune responses and maintaining immune tolerance. The imbalance between these two cell subsets could contribute to the chronic inflammation and abnormal sweating characteristic of PFH. Therefore, targeting the Th17/Treg axis could represent a potential therapeutic approach for managing PFH in clinic.

To validate the clinical relevance of these findings and evaluate the therapeutic potential of immunomodulation, we established a pilocarpine-induced mouse model of hyperhidrosis and assessed the effects of SR2211, a selective RORγ antagonist (MedChemExpress, Cat# HY-16998, purity 99.67%) with low reported off-target activity against RORα, RORβ, and kinases such as TRK and BRAF. Importantly, serum acetylcholine levels, a key neurotransmitter in sweat gland activation, were also markedly decreased. These findings indicate that SR2211 effectively suppresses sweat hypersecretion *in vivo*. Beyond functional improvement, SR2211 also alleviated Th17-associated inflammation in hyperhidrosis mice. The proportion of Th17 lymphocytes in peripheral blood was reduced, and ELISA results confirmed significant decreases in serum IL-17 and IL-6 levels following SR2211 treatment. Moreover, Western blot analysis revealed reduced IL-17A expression in sweat gland tissues. These results are consistent with our clinical observations and support the specificity of SR2211 in targeting RORγ, although residual off-target effects cannot be completely excluded. These results are consistent with our clinical observations in PFH patients and provide *in vivo* evidence that inhibition of RORγ activity can mitigate both hyperhidrosis symptoms and associated immune activation.

While the current study highlights the Th17/Treg imbalance in PFH pathophysiology, PFH is a complex condition potentially influenced by additional mechanisms, such as autonomic nervous system dysfunction, genetic predispositions, and environmental triggers (30). Exploring how these factors interact with or contribute independently to immune dysregulation could provide a more comprehensive view of PFH. Additionally, including insights from related studies on other immune or neural pathways could contextualize the observed imbalance within a multifactorial framework. This broader perspective might also suggest novel therapeutic targets beyond immunomodulation, such as interventions targeting neural-immune crosstalk or stress-related pathways.

Several limitations should be acknowledged to ensure a comprehensive interpretation of the study’s findings. First, the study included a relatively small number of participants, with only 60 patients in each PFH subtype group. A larger sample size would enhance the statistical power of the analysis and increase the generalizability of the findings. Second, the study design appears to be cross-sectional, which limits the ability to establish causal relationships between the Th17/Treg imbalance and PFH. Longitudinal studies with follow-up assessments would provide better insights into the dynamic changes of Th17/Treg balance over time and its potential role in disease progression. Longitudinal studies would be necessary to clarify whether the observed immune imbalance precedes or follows disease onset. Third, the manuscript does not provide mechanistic insights into why the Th17/Treg balance is altered in PFH. More detailed investigations, such as exploring the role of specific cytokines, cellular interactions, and signaling pathways, would strengthen the understanding of the underlying immune dysregulation. Fourth, the potential factors such as lifestyle (e.g., smoking, alcohol consumption) and socioeconomic variables that could influence immune markers were not considered, which might influence the immune markers. Last, the study population is region-specific, and the findings may not be universally applicable due to potential variations in immune profiles influenced by ethnic or environmental factors. Addressing these limitations in future research will help to further elucidate the role of the immune system in PFH and provide more comprehensive insights into potential treatment avenues.

## Conclusions

In conclusion, our study highlights the dysregulation of Th17/Treg cell balance in PFH patients and provides further evidence for the involvement of the immune system in the pathogenesis of this condition. The observed alterations in Th17/Treg cell ratio and cytokine profile, as well as the dysregulated expression of key transcription factors in sweat gland tissues, shed light on potential molecular targets for future therapeutic interventions. Importantly, *in vivo* validation using a mouse model demonstrated that SR2211, a selective RORγ antagonist, effectively alleviates both sweat hypersecretion and Th17-related inflammation. These findings support the potential of RORγ-targeted immunomodulation as a promising therapeutic strategy for PFH. However, further research is needed to elucidate the underlying mechanisms and evaluate the clinical applicability of targeting the Th17/Treg axis in the management of PFH.

## Data Availability

The original contributions presented in the study are included in the article/**Supplementary Material**. Further inquiries can be directed to the corresponding authors.
