# Supplementary material for: Altered Th17/Treg balance and therapeutic targeting of RORγ in primary focal hyperhidrosis
Source: Front Immunol. 2025 Oct 17;16:1656632. doi: 10.3389/fimmu.2025.1656632 (PMC12575253; doi:10.3389/fimmu.2025.1656632)
Supplement: Supplementary file 1 [file Table1.docx]

Supplementary materials


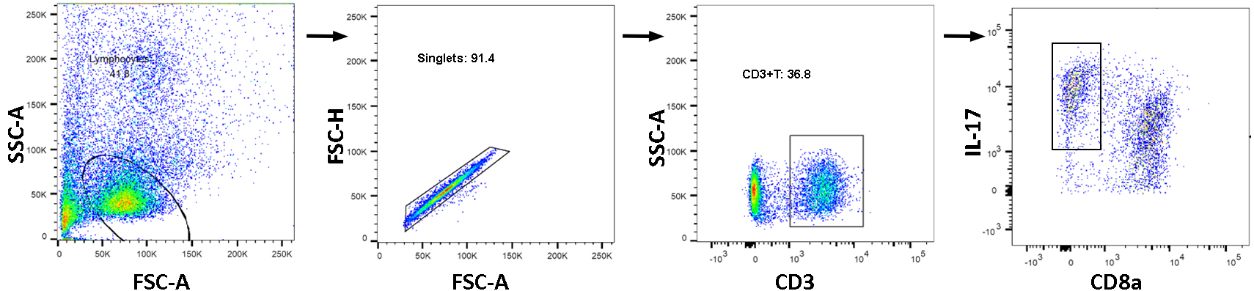


Figure S1. A representative gating strategy for screening of Th17 lymphocyte subpopulations in peripheral blood from patients with primary focal hyperhidrosis.


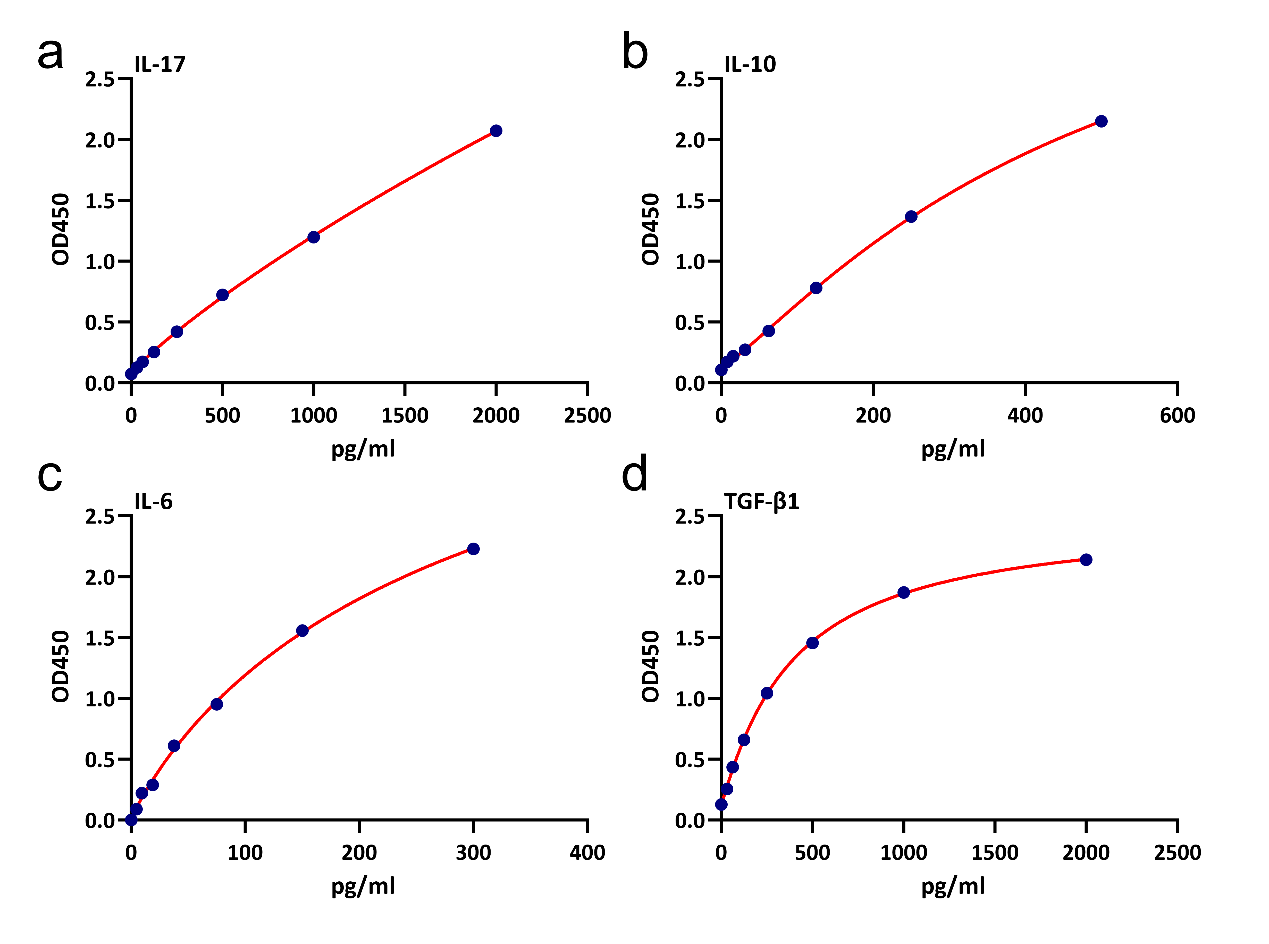


Figure S2. The standard curves used to calculate cytokine concentrations in the ELISA analysis. Formula for IL-17 concentration: X = 26523 × [(21.26 - Y)/(Y - 0.06906)]^(1/0.8748); for IL-10: X = 493.2 × [(4.137 - Y) / (Y - 0.1309)]^(1/1.197); for IL-6: X = 313.2 × [(4.550 - Y) / (Y - 0.003129)]^(1/0.9100); for TGF-β1: X = 387.2 × [(2.490 - Y) / (Y - 0.1246)]^(1/1.073). Where X is the concentration of indicated calculate cytokine in the sample (unit: pg/ml for all), Y is the measured OD450 absorbance value of the sample. The numerical constants in each formula are the 4PL parameters derived from nonlinear regression fitting of the corresponding standard curve.
